# Comparison of Different HIV-1 Resistance Interpretation Tools for Next-Generation Sequencing in Italy

**DOI:** 10.3390/v16091422

**Published:** 2024-09-06

**Authors:** Daniele Armenia, Luca Carioti, Valeria Micheli, Isabella Bon, Tiziano Allice, Celestino Bonura, Bianca Bruzzone, Fiorenza Bracchitta, Francesco Cerutti, Giovanni Maurizio Giammanco, Federica Stefanelli, Maria Addolorata Bonifacio, Ada Bertoli, Marialinda Vatteroni, Gabriele Ibba, Federica Novazzi, Maria Rosaria Lipsi, Nunzia Cuomo, Ilaria Vicenti, Francesca Ceccherini-Silberstein, Barbara Rossetti, Antonia Bezenchek, Francesco Saladini, Maurizio Zazzi, Maria Mercedes Santoro

**Affiliations:** 1 Departmental Faculty, UniCamillus, Saint Camillus International University of Health Sciences, 00131 Rome, Italy; 2 Department of Experimental Medicine, University of Rome “Tor Vergata”, 00133 Rome, Italy; 3 Laboratory of Clinical Microbiology, Virology and Bioemergencies, ASST Fatebenefratelli Sacco-University of Milan, 20157 Milan, Italy; 4 Microbiology Unit, IRCCS Azienda Ospedaliero-Universitaria di Bologna, 40138 Bologna, Italy; 5 Laboratory of Microbiology and Virology, Amedeo di Savoia Hospital, 10149 Turin, Italy; 6 Dipartimento di Promozione della Salute, Materno-Infantile, di Medicina Interna e Specialistica di Eccellenza “G. D’Alessandro” (PROSAMI), Azienda Ospedaliera Universitaria Policlinico “P. Giaccone”-University of Palermo, 90127 Palermo, Italy; 7 Hygiene Unit, Ospedale Policlinico San Martino, 16132 Genoa, Italy; 8 Section of Experimental and Clinical Pathology, Department of Precision and Regenerative Medicine and Jonic Area, University of Bari, 70121 Bari, Italy; 9 Virology Unit, Polyclinic of “Tor Vergata”, 00133 Rome, Italy; 10 Virology Unit, AOU Pisana, 56126 Pisa, Italy; 11 Microbiology and Virology Unit, Diagnostic Department, AOU Sassari, 07100 Sassari, Italy; 12 Department of Medicine and Technological Innovation, University of Insubria, 21100 Varese, Italy; 13 Microbiology and Virology Unit, Policlinico Riuniti Foggia Hospital, 71121 Foggia, Italy; 14 U.O.C. Microbiologia e Virologia, P.O. “D.Cotugno”-AO dei Colli, 80100 Napoli, Italy; 15 Department of Medical Biotechnologies, University of Siena, 53100 Siena, Italy; 16 Infectious Disease Department, USL SUDEST, Toscana, Misericordia Hospital, 58100 Grosseto, Italy; 17 IPRO-InformaPRO S.r.l., 00152 Rome, Italy; 18 EuResist Network GEIE, 00152 Rome, Italy

**Keywords:** next-generation sequencing, HIV drug resistance, HIV-1 subtype, viremia, minority variants, bioinformatic interpretation tools

## Abstract

Background: Next-generation sequencing (NGS) is gradually replacing Sanger sequencing for HIV genotypic drug resistance testing (GRT). This work evaluated the concordance among different NGS-GRT interpretation tools in a real-life setting. Methods: Routine NGS-GRT data were generated from viral RNA at 11 Italian laboratories with the AD4SEQ HIV-1 Solution v2 commercial kit. NGS results were interpreted by the SmartVir system provided by the kit and by two online tools (HyDRA Web and Stanford HIVdb). NGS-GRT was considered valid when the coverage was >100 reads (100×) at each PR/RT/IN resistance-associated position listed in the HIVdb 9.5.1 algorithm. Results: Among 629 NGS-GRT, 75.2%, 74.2%, and 70.9% were valid according to SmartVir, HyDRA Web, and HIVdb. Considering at least two interpretation tools, 463 (73.6%) NGS-GRT had a valid coverage for resistance analyses. The proportion of valid samples was affected by viremia <10,000–1000 copies/mL and non-B subtypes. Mutations at an NGS frequency >10% showed fair concordance among different interpretation tools. Conclusion: This Italian survey on NGS resistance testing suggests that viremia levels and HIV subtype affect NGS-GRT coverage. Within the current routine method for NGS-GRT, only mutations with frequency >10% seem reliably detected across different interpretation tools.

## 1. Introduction

Dramatic advances have been made in HIV management, thanks to the continuous improvement of antiretroviral therapy (ART) and extensive use of resistance testing from the time when treatment starts, and beyond [1,2]. Information about drug resistance helps in selecting more effective antiretroviral regimens and contributing to high rates of virologic success. Genotypic resistance testing (GRT) through Sanger bulk sequencing of plasma HIV RNA has been long and effectively supporting ART. However, this procedure only provides information on the tip of the iceberg, allowing the detection of resistant variants with >15–20% frequency [3,4,5]. The assessment of low-abundance drug-resistance mutations is now possible through Next-Generation Sequencing (NGS) approaches that are becoming affordable in terms of costs and turnaround time [6,7]. Moreover, several studies have shown that NGS-GRT is highly concordant with Sanger sequencing at a 20% threshold [8,9,10,11,12,13,14]. For these reasons, NGS-GRT is gradually replacing Sanger sequencing, but harmonization is lagging behind due to the heterogeneity of currently available lab platforms and NGS data analysis. In this context, virology laboratories are making a lot of effort to adapt their sequencing routine to the new technologies and interpret NGS data to provide an understandable clinical report. However, several issues still need to be addressed including the harmonization of NGS-based HIV drug resistance testing protocols and subsequent data processing and reporting, both of which may benefit from improved automation to minimize artificial errors. While Sanger sequences can be easily generated and analyzed by drug resistance interpretation algorithms such as the Stanford HIV Drug Resistance Database HIVdb algorithm (https://hivdb.stanford.edu/, accessed on 31 January 2024), the harmonization of NGS-GRT is more complex and includes three main steps: (1) wet-lab steps to generate PCR amplicons that cover the pol region and prepare NGS libraries; (2) loading products on NGS platforms; and (3) bioinformatics pipelines, which convert NGS data into user-interpretable HIV drug resistance results [15,16,17]. For the third step, several freely available web tools or standalone software embedded with commercial NGS kits are currently available, providing a straightforward assessment of resistance without any bioinformatics skills [10,18]. Among them, the NGS adaptation of HIVdb and HyDRA Web are well-consolidated free web tools widely used both for research and diagnostic purposes [13,19]. Among standalone tools, SmartVir is provided with the AD4SEQ HIV-1 Solution v2 kit, the most widely used commercial kit for HIV-1 NGS-GRT in Italy (https://www.arrowdiagnostics.it/, accessed on 31 January 2024). To our knowledge, no data on the concordance of NGS-GRT interpretation are available on a large scale. This work aimed at comparing HIVdb, HyDRA Web, and SmartVir for the analysis of NGS-GRT data obtained from a large dataset from Italian real-life settings.

## 2. Materials and Methods

### 2.1. Study Design

This is an observational study conducted on NGS data retrieved from 11 Italian virology laboratories, involved in the Italian collaborative HIV NGS Network created within the ARCA cohort (https://db.dbarca.net/, accessed on 31 January 2024) to share information, protocols, and data for improving the harmonization of NGS usage in routine HIV diagnostics. At present, the network connects 36 Italian centers involved in HIV GRT for both diagnostic and research.

All NGS-GRT were performed using plasma HIV-1 RNA samples of viremic people living with HIV (PLWH) processed by using the commercial kit AD4SEQ HIV-1 Solution v2 (Arrow Diagnostics S.r.l., Genova, Italy). According to the documentation provided by the manufacturer, this CE-IVD kit can reliably detect mutations (associated or not with drug resistance) in samples with viremia values >500 copies/mL for the diagnostic routine in different HIV-1 viral subtypes. For each NGS-GRT, information about contextual viremia, treatment status (ART-naïve or -experienced), and the NGS platform used (Illumina MiSeq or iSeq100) were retrieved. NGS data were interpreted through the standalone software provided with the commercial kit (SmartVir) and the two HyDRA Web and HIVdb free online tools.

### 2.2. RNA Extraction

All blood samples were centrifuged at 2400× *g* for 15 min at 4 °C for plasma separation. After centrifugation, RNA was extracted using different extraction kits (ELITE InGenius SP200/SP1000 kit, ELITechGroup; QIAamp UltraSens Virus Kit, QIAGEN; NucliSENS^®^ easyMag^®^/eMAG^®^, bioMèrieux), according to the manufacturer’s recommendations. The extraction and elution volumes were adjusted according to the viremia levels of samples: <1000 copies/mL (ultracentrifugation for 2 h at 4 °C—input volume of 400 μL^−1^ mL eluted in 50 μL), 1000–5000 (input volume of 400 μL^−1^ mL eluted in 50 μL), and >5000 (input volume of 200 μL^−1^ mL eluted in 50–100 μL).

### 2.3. Library Preparation for Illumina iSeq100/MiSeq Platforms

The amplicon-based library was prepared according to the manufacturer’s recommendations, allowing us to amplify and sequence protease (PR: 1–99 aa), reverse transcriptase (RT: 1–440 aa), and integrase (INT: 1–288 aa) regions, with a theoretical analytical sensitivity of 500 copies/mL. The final pool was loaded with 10% PhiX control at 9 pM on the MiSeq V2 reagent kit 500-cycle cartridge and MiSeq Reagent Nano kit v2 flow cell (Illumina Inc., San Diego, CA, USA), or at 110 pM on iSeq100 i1 reagent 300-cycle cartridge v2 (Illumina Inc., San Diego, CA, USA).

### 2.4. Sequence Data Analysis and Coverage Evaluation

The raw FastQ files obtained after sequencing were analyzed using the standalone software SmartVir (SmartSeq s.r.l., Alessandria, Italy) and the web tools HyDRA Web (Ver v1.7.0; available at https://hydra.canada.ca/pages/home, accessed on 31 January 2024) and HIVdb (ver 9.5.1; available at: https://hivdb.stanford.edu/hivdb/by-reads/, accessed on 31 January 2024) set at default parameters for filtering low-quality reads and at 1% cut-off for variant detection. Subtype was assessed according to the HIVdb ver 9.5.1 algorithm. Frequency cut-offs of 5% and 20% were also evaluated. Coverage >100 reads (100×) per position was considered the minimum cut-off for the valid detection of HIV drug resistance as previously reported for HIV NGS-GRT [5,20] and according to manufacturer instructions. Specifically, an NGS-GRT was considered valid for resistance analyses when 100× at each PR/RT/IN resistance-associated position listed in the HIVdb algorithm ver 9.5.1 was obtained. The reliability rate ([number of valid NGS-GRT] × 100/[total NGS-GRT]) was calculated for each tool and for at least two tools. The reliability rate was stratified according to subtype, viremia, platform used, and treatment status. In particular, viremia levels were stratified according to the following strata: ≤1000; 1001–10,000; 10,001–100,000; 100,001–1,000,000; >1,000,000 HIV-1 RNA copies/mL. Predictors of reliability for resistance interpretation were assessed through univariable and multivariable logistic regression.

### 2.5. Resistance Analysis and Variant Detection Concordance

**Figure 1:**
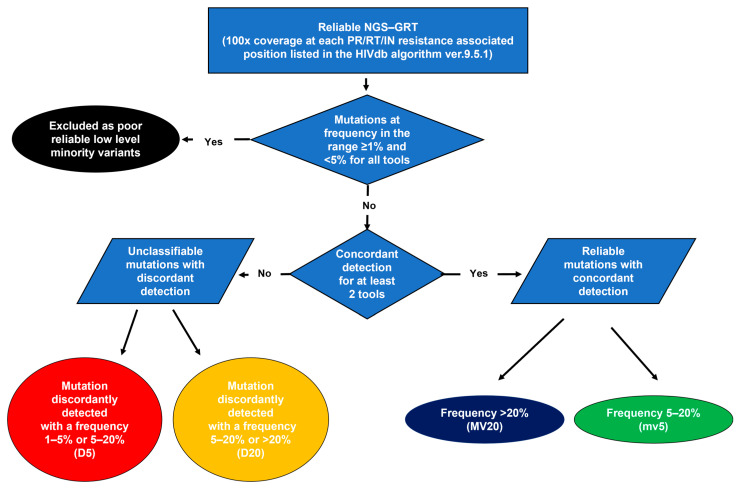
Flow chart for the evaluation of concordance among tools for HIV-1 NGS data interpretation in resistance assessment.

The flow chart for resistance analysis is reported in Figure 1. All the substitutions detected at resistance-associated positions with a frequency >1% were evaluated. Mutations were stratified according to the following frequency detection groups: (i) frequency ranging from 1% to 5%: low-level minority variants; (ii) frequency ranging from 5% to 20%: minority variants; and (iii) frequency >20%: majority variants. The detection of unusual mutations (defined as having a prevalence <0.01% in HIV-1 group M sequences) according to the HIVdb algorithm or stop codons was considered as part of the assessment of sequencing accuracy. Mutations concordantly detected from all the interpretation tools as low-level minority variants were excluded and not considered valid for resistance interpretation according to already published studies on NGS data [19,21]. To assess the concordance in resistance detection among different interpretation systems, the frequency of each mutation was considered consistent when it fell within the same frequency detection group across the interpretation tools. Concordance was assessed overall and per each position of PR, RT, and IN regions analyzed.

### 2.6. Statistical Analyses

Differences between dichotomous or categorical variables were assessed using Chi-Squared or Fisher exact tests as appropriate. Differences between continuous variables were determined with the Mann–Whitney or Kruskal–Wallis test as appropriate. Univariable and multivariable logistic regression models were built to assess whether subtype, viremia levels, NGS platform, and treatment status were predictors of coverage reliability. The coefficient of variation (CV), defined as the ratio of the standard deviation to the mean of mutation’s frequency, was calculated to estimate the variability in detecting mutations across interpretation tools. The maximum frequency of mutations with discordant frequency detection group was calculated and the third quartile (3rdQ) of the distributions was considered the threshold of frequency above which misclassifications are less likely to be present.

## 3. Results

### 3.1. Samples’ Characteristics and Coverage Reliability Rate

The characteristics of the 629 samples analyzed are reported in Table 1. Most of them had a contextual viremia >1000 copies/mL (92.5%) and were processed through the iSeq100 platform (77.1%). A considerable proportion of samples were from individuals infected with HIV-1 non-B subtypes (41.2%).

**Figure 2:**
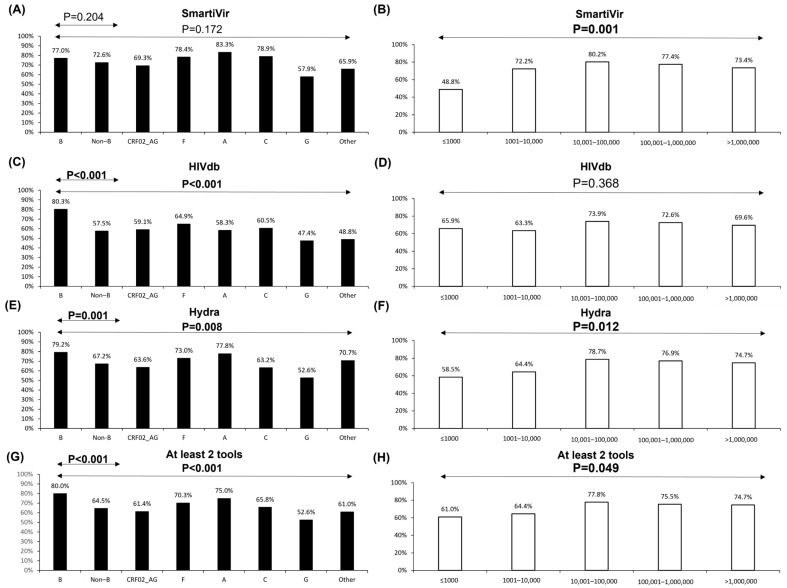
Proportion of samples with valid coverage for resistance evaluation obtained through different NGS data interpretation tools, according to subtypes and contextual viremia levels. Bars represent the proportion of samples with 100× coverage among all PR/RT/IN positions associated with resistance according to the Stanford drug resistance algorithm (HIVdb 9.5.0). Black and white bars represent proportions according to subtypes and viremia levels, respectively. (**A**,**B**) SmartVir standalone tool; (**C**,**D**) free web tool HIVdb; (**E**,**F**) free web tool HyDRA Web; (**G**,**H**) at least 2 tools among SmartVir, HyDRA Web, and HIVdb.

The reliability rate was 75.2%, 74.2%, and 70.9 according to SmartVir, HyDRA Web, and HIVdb. Four-hundred and sixty-three (73.6%) NGS-GRT had valid coverage for resistance analyses with at least two interpretation tools. These proportions were affected by HIV-1 subtype and viremia (Figure 2). Namely, the two online interpretation tools were significantly affected by non-B subtypes (Figure 2C,E,G) while subtype G was associated with the lowest rate of coverage reliability with all the interpretation tools. A slight decrease in the coverage reliability rate (below 73%) was observed for all three tools even at plasma HIV-1 RNA levels <10,000 copies/mL (Figure 2B,D,F,H). However, the coverage reliability rate considerably decreased at viremia levels ≤1000 copies/mL, specifically for HyDRA Web and SmartVir tools (Figure 2B,F).

When evaluating valid coverage for resistance interpretation by at least two tools, multivariable logistic regression analyses showed that viremia levels below 10,000 copies /mL (and especially below 1000 copies/mL) and subtypes CRF02_AG, G, and/or heterogeneous recombinant forms were negatively associated with a valid coverage for resistance analyses (Table 2). When each tool was evaluated separately, results varied and only the subtype transversally affected coverage reliability (Table 2).

### 3.2. Resistance Interpretation

Among 463 GRT with valid coverage in PR/RT/IN for at least two tools, 9596 mutations were detected. Of these, 7483 (78.0%) were low-level minority variants. Concerning unusual mutations, among the 626 mutations identified, the highest proportion (561, 89.6%) was detected as low-level minority variants, followed by discordant minority variants ranging from 5–20% (55, 8.8%) and majority variants (10, 1.6%). They were never found among mutations concordantly detected by at least two tools (0%, *p* < 0.001).

Overall, excluding low-level minority variants and unusual mutations, 2049 (690 in PR; 768 in RT; 591 in IN) mutations with a frequency >5% for at least one tool were identified, most concordantly detected with a median (IQR) CV of 1.4% (0.5–8.2%). Specifically, 1739 (84.9%) mutations were concordantly detected by at least two tools (majority variants: 64.0%; minority variants: 20.9%). Discordant mutations (N = 310, 15.1%) were mostly minority variants (frequency range 5–20%: 12.4%; frequency >20%: 2.6%) and were more likely observed in the IN region. In fact, the highest median CV was observed among IN mutations (median [IQR] CV IN: 6.1% [1.2–20.4%]; RT: 1.2% [0.4–6.6%]; PR: 0.7% [0.4–2.9%], *p* < 0.001).

**Figure 3:**
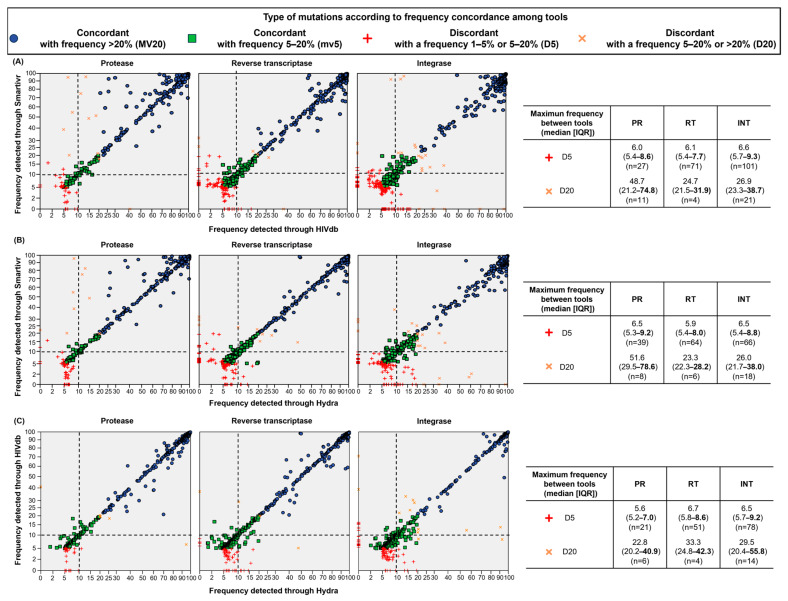
Concordance in detecting mutations between different NGS resistance interpretation tools. (**A**) Comparison between SmartVir and HIVdb; (**B**) comparison between SmartVir and HyDRA Web; (**C**) comparison between HyDRA Web and HIVdb. Each symbol represents a substitution detected with a valid coverage per each tool. Blue and green symbols represent mutations concordantly detected as majority (frequency >20%) and minority variants (frequency 1–5%), respectively. Red crosses represent mutations discordantly detected as low-level minority mutations (frequency 1–5%) or minority mutations (5–20%). Orange crosses represent mutations discordantly detected as minority mutations (frequency 5–20%) or majority mutations (5–20%). In the table on the right of scatter plots, the distribution of maximum frequency of discordant mutations detected between tools is reported; the third quartile of the distribution is highlighted in bold. Dotted lines represent 10% of frequency cut-off.

Mutation reports were then evaluated by comparing each tool to the other (Figure 3). Good concordance was observed above a 10% frequency. Discordant minority variants were mainly detected below 10% frequency regardless of the tool used. In general, discordant majority variants were less likely to be observed.

### 3.3. Concordance in Detecting Mutations at Resistance-Associated Positions of PR/RT/IN

**Figure 4:**
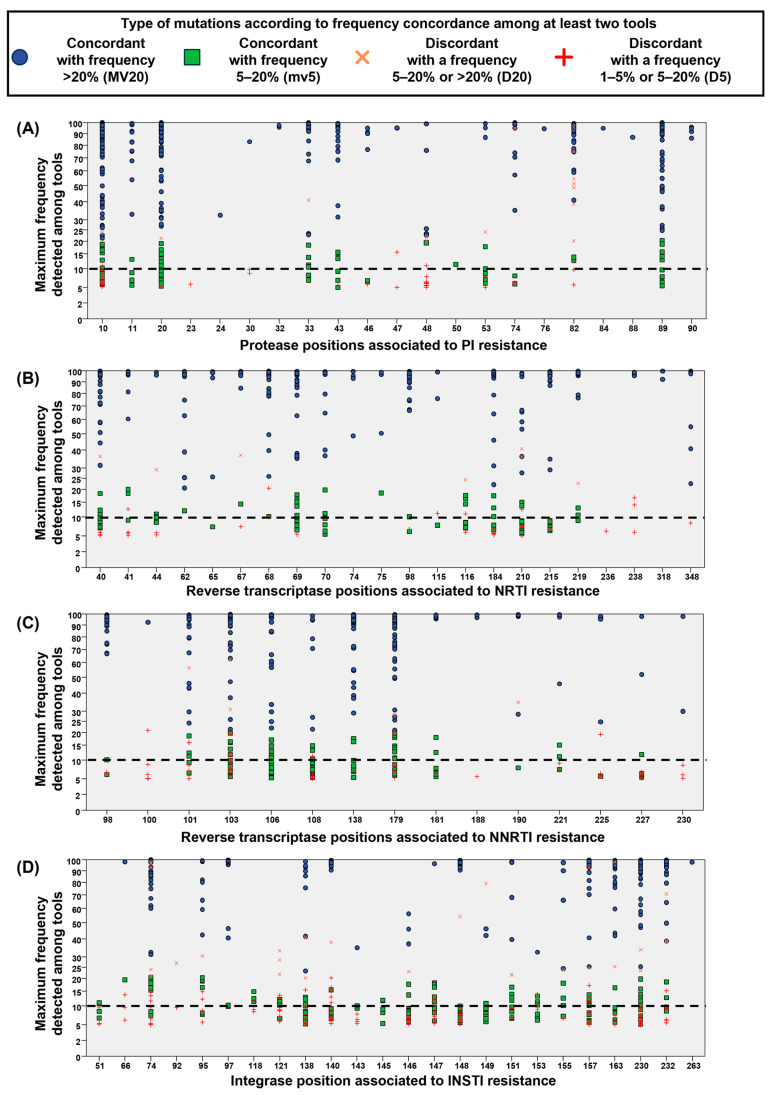
Concordance in detecting mutations among at least two NGS resistance interpretation tools at each position associated with resistance spanning PR/RT/IN. (**A**) Protease positions associated with resistance to PI. (**B**) Reverse transcriptase positions associated with resistance to NRTI. (**C**) Reverse transcriptase positions associated with resistance to NNRTI. (**D**) Integrase positions associated with resistance to INSTI. X axis represents the positions associated with drug resistance according to the Stanford drug resistance algorithm (HIVdb 9.5.0); Y axis represents the maximum frequency detected in the reports retrieved from the three interpretation tools. Each symbol represents a substitution detected with a valid coverage in at least two tools. Blue and green symbols represent mutations concordantly detected as majority (frequency > 20%) and minority variants (frequency 1–5%), respectively. Red crosses represent mutations discordantly detected as low-level minority mutations (frequency 1–5%) or minority mutations (5–20%). Orange crosses represent mutations discordantly detected as minority mutations (frequency 5–20%) or majority mutations (5–20%).

The frequency distribution of mutations detected at each resistance-associated position in the PR/RT/IN regions is reported in Figure 4. In the majority of positions, most mutations were concordantly detected. Concerning discordant mutations, the maximum frequency among tools of minority variants with discordant detection was rarely observed above 10%. In particular, discordant minority variants with a maximum frequency above 10% were found, especially in IN, as follows: (i) 4 spanning PR at positions 10, 47, 48, and 82 (Figure 4A); (ii) 17 spanning RT at positions 100, 101, 103, 106, 108, 179, 225, and 238; and (iii) 28 spanning IN at position 66, 74, 95, 118, 121, 138, 140, 147, 151, 153, 157, and 232 (Figure 4B–D). In general, discordant majority variants (orange crosses) were more rarely detected.

## 4. Discussion

Considering the ongoing transition from Sanger to NGS technologies for routine HIV-1 resistance testing, the collection and evaluation of information retrieved from NGS in real life is crucial. In the present study, for the first time at a nationwide level in Italy, a considerable amount of NGS data (including several HIV-1 non-B subtypes) retrieved from real life was collected and explored. Among the 629 NGS-GRT tested with three different interpretation tools, around 70–75% had valid coverage (at least 100×) to evaluate drug resistance mutations in the HIV-1 PR/RT/IN regions. The coverage rate observed in this study was similar to or even higher than that observed in other studies evaluating in-house or commercial kits [14,20,22].

A key question is whether around 75% of NGS-GRT success might be satisfactory in the clinical setting. Notably, certain HIV-1 subtypes and moderate-low viremia levels may affect this success rate. In fact, we found that subtype G and heterogeneous recombinant forms were associated with the lowest rate (<60%) of valid coverage regardless of the interpretation tool used. This was not observed in previous studies evaluating in-house or commercial NGS methods [20,23], likely because of the lower HIV-1 subtype heterogeneity among samples tested. Indeed, in the present study, more than 40% of clinical samples had non-B subtype strains, including around 10% of heterogeneous CRF. Thus, technical adjustments should be implemented to reduce the impact of genetic variability on the coverage issue.

Concerning viremia levels, a slight decrease in coverage reliability was generally observed at levels below 10,000 copies/mL, thus raising the concern about the proper identification of resistance mutations at viral loads commonly linked to viral failure during antiviral therapy and an increased likelihood of drug resistance emergence [24,25,26].

Nevertheless, a considerable decrease in the coverage reliability rate was observed for samples with contextual viremia below 1000 copies/mL and this has been emphasized by the inclusion of several samples with viremia levels below 500 copies/mL (median [IQR]: 276 [187–510] copies/mL), which is the analytical sensitivity threshold reported for the kit used in this study.

Taken together, these results confirm that NGS for routine HIV GRT is affordable even if there are still some issues related to HIV-1 non-B subtypes and low viral loads. Unfortunately, these limitations are clinically relevant given the increasing number of non-B subtypes in many geographic areas (including Italy) [27,28,29] and the frequent GRT requests for individuals with low-level viremia [30].

Another important aspect evaluated in this study is the concordance in detecting mutations using different NGS data processing tools. This is an important point considering that virologists may use multiple interpretation tools for making resistance reports, especially to verify the presence of resistance mutations as minority variants. Several studies suggest that minority variants with a frequency below 3–5% may result from sequencing artifacts [19,21]. Indeed, we observed the highest proportion of mutations recognized as unusual and/or affected by discordant detection at a frequency in the range of 1 to 5%, supporting the technical cut-off for HIV NGS-GRT at 5%. This finding is in line with the manufacturer’s recommendations about the frequency cut-off to be considered. However, at a frequency >5%, variation among tools in detecting minority variants remained quite common, affecting around 15% of the total number of mutations detected, particularly in the IN region. In fact, the highest median CV was observed for IN mutations and discordant mutations were observed widely across the IN region. Again, this is relevant considering that INSTI are the most currently used drugs, recommended as part of first-line regimens from guidelines [1,2], and uncertainty in detecting resistance minority variants might become challenging when making GRT reports. Despite this, most of the discordant mutations were generally detected below 10%. While this is important information to guide resistance interpretation among virology labs that use commercial kits, differences may arise with other NGS and analysis pipelines. For example, another study evaluating five NGS HIV drug resistance pipelines [17] concluded that the 2% threshold may be appropriate to detect resistance mutations with high specificity. Given the wide heterogeneity in wet-lab procedures, platforms, and interpretation systems, a universal technical cut-off may remain challenging to set.

This study might have some limitations. First of all, the real performance of the whole NGS-GRT system was not evaluated because we compared only the NGS data interpretation tools. Since all the samples evaluated were processed with the same commercial kit for NGS, the technical cut-offs proposed cannot be extrapolated to other commercial or in-house approaches. Finally, no information about the clinical role of minority mutations can be concluded considering the mere technical scope of the present study.

## 5. Conclusions

In conclusion, the usage of NGS-GRT in Italy is increasing, mostly based on the availability of a standardized kit. However, technical improvements are required. At present, a technical frequency cut-off of 10% should be advisable to process the NGS-GRT data and guide a cautious transition from the well-established Sanger sequencing to NGS in the clinical setting. Clearly, given the extraordinary pace of technical progress in the field, recommendations are subject to frequent updates to ensure that clinical applications benefit from the latest technology.

## Figures and Tables

**Table 1:** Sample characteristics.

Characteristics	Overall (n = 629)
**Age, years, Median (IQR)**	44 (34–53)
**Year of genotyping, n (%)**	
<2020	28 (4.4)
2020–2021	104 (16.5)
2022–2023	497 (79.1)
**Plasma HIV-1 RNA, log (copies/mL), Median (IQR)**	4.9 (4.2–5.6)
≤1000 copies/mL, n (%)	41 (6.5)
1001–10,000 copies/mL, n (%)	90 (14.3)
10,001–100,000 copies/mL, n (%)	207 (32.9)
100,001–1,000,000 copies/mL, n (%)	212 (33.7)
>1,000,000 copies/mL, n (%)	79 (12.6)
**HIV-1Subtype, n (%) ^1^**	
B	370 (58.8)
CRF02_AG	88 (14.0)
F	37 (5.9)
C	36 (5.7)
A	38 (6.0)
G	19 (3.8)
Others ^2^	41 (6.5)
**Treatment status, n (%)**	
Naive	313 (49.8)
Experienced	152 (24.2)
Unknown	164 (26.1)
**Platform used for NGS**	
iSeq100	485 (77.1)
MiSeq	144 (22.9)

^1^ Subtype assessed through HIVdb algorithm 9.5.1. ^2^ Other subtypes: B + F (N = 10), CRF01_AE (N = 9), B + G (N = 5), CRF18_cpx (N = 4), CRF06_cpx (N = 2), CRF09_cpx (N = 2), CRF25_cpx (N = 2), D (N = 2), A + G (N = 1), B + C (N = 1), B + CRF01_AE (N = 1), CRF12_BF (N = 1), CRF29_BF (N = 1).

**Table 2:** Factors associated with coverage reliability rate for resistance interpretation of NGS-GRT performed for clinical routine.

Variables	Odd Ratio to Have Reliable Coverage for Resistance Interpretation (100X Coverage at All PR/RT/IN Resistance Positions)															
	At Least 2 Tools				SmartVir				HIVdb				Hydra			
	Crude		Adjusted ^1^		Crude		Adjusted ^1^		Crude		Adjusted ^1^		Crude		Adjusted ^1^	
	OR (95% C.I.)	*p* Value	OR (95% C.I.)	*p* Value	OR (95% C.I.)	*p* Value	OR (95% C.I.)	*p* Value	OR (95% C.I.)	*p* Value	OR (95% C.I.)	*p* Value	OR (95% C.I.)	*p* Value	OR (95% C.I.)	*p* Value
**Subtype**																
B ^2^	1		1				1		1		1		1		1	
CRF02_AG	**0.4 (0.2–0.7)**	**<0.001**	**0.4 (0.2–0.6)**	**<0.001**	0.7 (0.4–1.1)	0.132	0.6 (0.3–1.0)	0.051	**0.4 (0.2–0.6)**	**<0.001**	**0.4 (0.2–0.6)**	**<0.001**	**0.5 (0.3–0.8)**	**0.002**	**0.4 (0.2–0.7)**	**0.001**
F	0.6 (0.3–1.3)	0.169	0.5 (0.3–1.2)	0.11	1.1 (0.5–2.5)	0.852	1.1 (0.5–2.5)	0.892	**0.5 (0.2–0.9)**	**0.032**	**0.5 (0.2–1)**	**0.043**	0.7 (0.3–1.5)	0.381	0.7 (0.3–1.4)	0.293
A	0.8 (0.3–1.7)	0.479	0.7 (0.3–1.6)	0.414	1.5 (0.6–3.7)	0.389	1.3 (0.5–3.4)	0.541	**0.3 (0.2–0.7)**	**0.003**	**0.3 (0.2–0.7)**	**0.003**	0.9 (0.4–2.1)	0.843	0.9 (0.4–2)	0.764
C	**0.5 (0.2–1)**	**0.045**	0.5 (0.2–1)	0.052	1.1 (0.5–2.5)	0.788	1.1 (0.5–2.7)	0.752	**0.4 (0.2–0.8)**	**0.006**	**0.4 (0.2–0.8)**	**0.013**	**0.5 (0.2–0.9)**	**0.027**	**0.4 (0.2–0.9)**	**0.025**
G	**0.3 (0.1–0.7)**	**0.007**	**0.3 (0.1–0.7)**	**0.005**	0.4 (0.2–1.1)	0.064	**0.4 (0.1–1.0)**	**0.040**	**0.2 (0.1–0.6)**	**0.002**	**0.2 (0.1–0.5)**	**0.001**	**0.3 (0.1–0.7)**	**0.010**	**0.3 (0.1–0.7)**	**0.009**
Others	**0.4 (0.2–0.8)**	**0.007**	**0.3 (0.2–0.7)**	**0.002**	0.6 (0.3–1.1)	0.116	**0.5 (0.2–0.9)**	**0.028**	**0.2 (0.1–0.5)**	**<0.001**	**0.2 (0.1–0.5)**	**<0.001**	0.6 (0.3–1.3)	0.215	0.5 (0.2–1.1)	0.079
**Platform used**																
Illumina Iseq100 ^2^	1		1		1				1		1		1			
Illumina Miseq	1.5 (0.9–2.3)	0.086	1.6 (1–2.5)	0.065	0.9 (0.6–1.4)	0.615	-	-	2.1 (1.3–3.3)	0.002	**2.1 (1.2–3.6)**	**0.008**	1.2 (0.8–1.9)	0.376	-	-
**Viremia (copies/mL)**																
≤1000	0.5 (0.3–1)	0.058	**0.4 (0.2–0.7)**	**0.005**	**0.3 (0.1–0.6)**	**<0.001**	**0.3 (0.1–0.5)**	**<0.001**	0.7 (0.4–1.5)	0.379	-	-	**0.4 (0.2–0.9)**	**0.016**	**0.3 (0.2–0.7)**	**0.004**
1001–10,000	0.6 (0.3–1)	0.052	**0.5 (0.3–0.9)**	**0.014**	0.8 (0.4–1.3)	0.341	0.7 (0.4–1.3)	0.279	0.7 (0.4–1.1)	0.108	-	-	**0.5 (0.3–0.9)**	**0.027**	**0.5 (0.3–0.9)**	**0.012**
10,001–100,000	1.1 (0.7–1.8)	0.577	1.1 (0.7–1.7)	0.821	1.2 (0.7–1.9)	0.478	1.2 (0.7–1.9)	0.559	1.1 (0.7–1.6)	0.769	-	-	1.1 (0.7–1.8)	0.647	1 (0.6–1.7)	0.901
100,001–1,000,000 ^2^	1		1		1		1		1				1		1	
>1,000,000	1 (0.5–1.7)	0.89	1 (0.5–1.8)	0.874	0.8 (0.4–1.5)	0.483	0.8 (0.4–1.4)	0.383	0.9 (0.5–1.5)	0.611	–	–	0.9 (0.5–1.6)	0.694	0.9 (0.5–1.6)	0.629
**Treatment status**																
cART naïve ^2^	1				1				**1**				**1**			
cART experienced	1.3 (0.8–2)	0.249	-	-	1.0 (0.6–1.5)	0.873	1.1 (0.7–1.9)	0.622	**1.7 (1.1–2.7)**	**0.015**	1.4 (0.9–2.2)	0.192	1.2 (0.8–1.9)	0.377	-	-
Unknown	1.3 (0.8–1.9)	0.31	-	-	**0.6 (0.4–1.0)**	**0.038**	0.7 (0.4–1.1)	0.101	**1.6 (1.0–2.4)**	**0.040**	0.9 (0.6–1.6)	0.829	1 (0.7–1.6)	0.885	-	-

^1^ Variables with *p* < 0.1 at univariable analyses were included in multivariable models. ^2^ Reference (dummy). Reported variables significantly associated with reliable coverage are in bold.

## Data Availability

The data presented in this study are available upon request from the corresponding author because they contain personal sensitive information subject to GDPR; the related privacy and ethical issues are managed by following the ARCA Board rules on sensitive data sharing for research purposes.
